# Quantification of the Animal Tuberculosis Multi-Host Community Offers Insights for Control

**DOI:** 10.3390/pathogens9060421

**Published:** 2020-05-28

**Authors:** Nuno Santos, Céline Richomme, Telmo Nunes, Joaquín Vicente, Paulo C. Alves, José de la Fuente, Margarida Correia-Neves, María-Laura Boschiroli, Richard Delahay, Christian Gortázar

**Affiliations:** 1 CIBIO/InBio, Research Center in Biodiversity and Genetic Resources, University of Porto, Campus of Vairão, 4485-661 Vila do Conde, Portugal; pcalves@fc.up.pt; 2 ANSES Nancy Laboratory for Rabies and Wildlife, 54220 Malzéville, France; Celine.RICHOMME@anses.fr; 3 CIISA, Centro de Investigação Interdisciplinar em Sanidade Animal, University of Lisbon, 1300-477 Lisbon, Portugal; pinanunes@gmail.com; 4 SaBio Instituto de Investigación en Recursos Cinegéticos IREC (UCLM & CSIC), 13005 Ciudad Real, Spain; joaquin.vicente@uclm.es (J.V.); JosedeJesus.Fuente@uclm.es (J.d.l.F.); christian.gortazar@uclm.es (C.G.); 5 Dep. Biology, Faculty of Sciences, University of Porto, 4169-007 Porto, Portugal; 6 Wildlife Biology Program, University of Montana, Missoula, MO 59812, USA; 7 Oklahoma State University, Stillwater, OK 74078, USA; 8 ICVS, Life and Health Sciences Research Institute, 4710-057 Braga, Portugal; mcorreianeves@med.uminho.pt; 9 ICVS/3B’s PT Government Associate Laboratory, 4710-057 Braga, Portugal; 10 ANSES Laboratory for Animal Health, Tuberculosis National Reference Laboratory, University Paris-Est, 94000 Maisons-Alfort, France; Maria-laura.BOSCHIROLI@anses.fr; 11 National Wildlife Management Centre, Animal and Plant Health Agency, Gloucestershire GL10 3UJ, UK; Dez.Delahay@apha.gov.uk

**Keywords:** mycobacterium bovis, disease eradication, livestock, wild animals, stochastic models

## Abstract

Animal tuberculosis (TB) is a multi-host zoonotic disease whose prevalence in cattle herds in Europe has been increasing, despite a huge investment in eradication. The composition of the host community is a fundamental driver of pathogen transmission, and yet this has not been formally quantified for animal TB in Europe. We quantified multi-host communities of animal TB, using stochastic models to estimate the number of infected domestic and wild hosts in three regions: officially TB-free Central–Western Europe, and two largely TB-endemic regions, the Iberian Peninsula and Britain and Ireland. We show that the estimated number of infected animals in the three regions was 290,059–1,605,612 and the numbers of infected non-bovine domestic and wild hosts always exceeded those of infected cattle, with ratios ranging from 3.3 (1.3–19.6):1 in Britain and Ireland to 84.3 (20.5–864):1 in the Iberian Peninsula. Our results illustrate for the first time the extent to which animal TB systems in some regions of Europe are dominated by non-bovine domestic and wild species. These findings highlight the need to adapt current strategies for effective future control of the disease.

## 1. Introduction

Animal tuberculosis (TB) is a zoonotic disease caused by infection with mycobacteria of the *Mycobacterium tuberculosis* complex, whose host range includes many wild and domestic mammal species [1]. The epidemiology of such multi-host diseases is more complex and hence less predictable than for single-host diseases, and it follows that their control is particularly challenging [2]. It is only possible to eradicate such pathogens if control encompasses all the reservoir(s), which are defined as epidemiologically connected populations in which the pathogen can be maintained and from which infection is transmitted to the target population [3].

Evidence for TB maintenance in multi-host systems in Europe arises from molecular epidemiological studies demonstrating inter-species transmission between cattle, non-bovine domestic species, and wildlife [4,5]; observational studies reporting TB in domestic and wild animals as a risk factor for cattle [6,7]; and case reports of inter-species transmission [8]. Experimental studies have demonstrated the inter-species transmission of *M. bovis* under controlled and natural conditions [9,10]. In multi-host systems, sympatric hosts might differentially contribute to disease maintenance, and thus the composition of the host community is a fundamental driver of pathogen transmission [2,11].

Animal TB has been subject to eradication programs in many countries over public health and livestock trade concerns. Programs based on test and cull strategies and abattoir surveillance, exclusively targeting cattle, achieved significant success in the late 20th century [12]. Hence, all but 10 European Union (EU) countries were classified as Officially Tuberculosis-Free (OTF), as >99.9% of their cattle herds were free from disease for at least 6 consecutive years [13]. However, since then progress has stalled, with herd prevalence in the EU increasing from 0.59% in 2010 to 0.86% in 2017 [14], despite combined national and EU budgets for TB eradication in cattle of over 1 billion € during that time [15]. This has been partly attributed to the emergence of wildlife reservoirs and growing evidence that non-bovine domestic species might also play a significant role in disease persistence [12,16,17]. 

Animal TB persists in multi-host systems [2] in some parts of Europe, presenting significant challenges for disease management, but the composition and relative sizes of the infected host communities have never been explicitly quantified. We provide a new perspective on the epidemiology of TB in such multi-host systems by estimating the composition of the infected community of wildlife and domestic species, using stochastic modeling of data from three epidemiologically distinct European regions (OTF Central-Western Europe; non-OTF Britain and Ireland, and the non-OTF Iberian Peninsula) as case studies. Some areas in Britain (Scotland and Isle of Man) and the Iberian Peninsula (Algarve) are classified as OTF, but these represent <30% of the area of each region.

## 2. Results

Stochastic models allow us to incorporate the variability in parameters used to estimate the number of TB-infected animals as prior distributions [18]. The prior distributions of the apparent prevalence, sensitivity, and specificity of the diagnostic tests and host abundance were based on data obtained from studies published mostly (81.1%) since 2009 (Supplementary Materials Tables S1–S10). We used this information as a broad indicator of the epidemiological situation in the study regions in any given year from 2009 to 2018, assuming constant prevalence, abundance, and test performance within the timeframe of the data used for each region.

**Figure 1:**
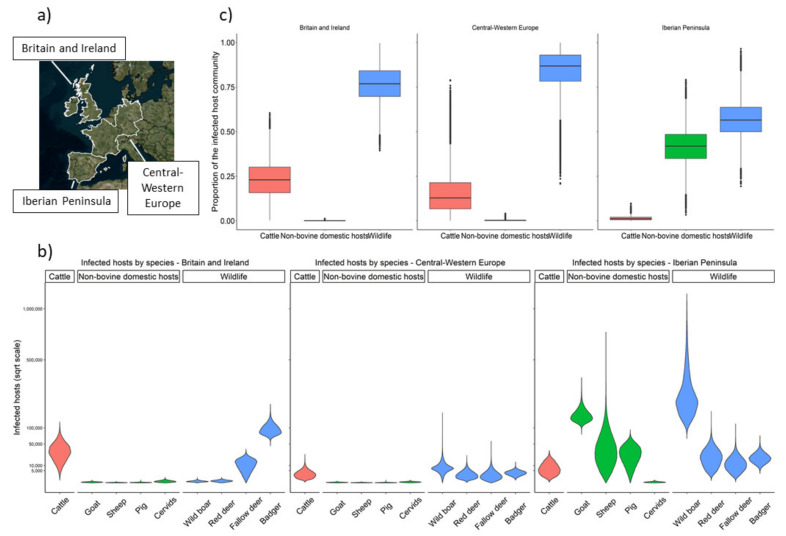
Estimated number of infected hosts in the three geographical regions. (**a**) Map of the European study regions; (**b**) Violin plots of the posterior distribution of the number of infected hosts by region (on the square root scale) with an equal width assigned to each distribution; (**c**) Boxplots of the proportion of cattle, non-bovine domestic hosts, and wildlife in the infected community by region.

The number of individual infected hosts in the three case study regions (Figure 1a) at any given time point was estimated at 290,059–1,605,612. Among these, wild animals are most prominent (178,678–1,043,651), followed by non-bovine domestic animals (109,751–440,539), and cattle (1,630–121,422) (Table 1).

The estimated composition of the community of infected hosts differs widely amongst the three regions (Figure 1b,c). In Britain and Ireland, it is dominated by badgers and cattle (Simpson’s index D = 0.536), while in Central–Western Europe, it is more diverse (D = 0.328), being dominated by wildlife and cattle. In the Iberian Peninsula (D = 0.318), the community of infected hosts is split more equally between wildlife (notably wild boar) and non-bovine domestic species. Non-bovine domestic and wild species constitute the majority of the community of infected animals in all three study regions, despite large regional variations (Table 2).

## 3. Discussion

Our study describes the multi-host characteristics of animal TB in Europe, and in the regions examined, it suggests that the majority of infected hosts are non-bovine. The extent to which these systems seem to be dominated by non-bovine species (Table 2) is surprising and has implications for the successful control of infection in cattle. Our findings imply that interventions to manage transmission from non-bovine domestic and wildlife host populations may be necessary to achieve the effective control of TB in cattle. Additional information on the transmission routes and roles of each host species in transmission to cattle [2,3] in these systems will be necessary to assess which approaches are likely to be most successful.

The relatively small community of infected hosts estimated for Central–Western Europe was expected given its OTF status, which was achieved at a time when multi-host TB systems were not yet known in continental Europe [13]. On the other hand, the regions that did not achieve OTF status until the early 2000s now face an established multi-host community whose composition and size depend on regional factors. The emergence of the wild boar as a significant TB host in mainland Europe [1], particularly in the Iberian Peninsula, is likely related to substantial population growth [19,20]. Susceptibility to infection and the locally intensive management of wild boar populations have resulted in regionally high apparent prevalence (estimated at 0.630, CI_95_ 0.351–0.939, in the endemic region in Spain). Furthermore, a proportion of infected animals shed the pathogen simultaneously via several routes (0.13, CI_95_ 0.06–0.27 [21]), and evidence suggests that infection is maintained in wild boar populations [19]. 

In Britain and Ireland, the most important wild host is the badger, which has also seen a significant increase in abundance over recent decades [22]. We estimated the true prevalence of infection in badgers across the high risk and endemic areas of the United Kingdom to be 0.21 (CI_95_ 0.15–0.33) (Supplementary Materials). Post-mortem investigations have revealed progressive disease in some infected badgers with the potential to shed bacteria [23], and longitudinal sampling of live animals suggests the existence of a sub-group that persistently shed from multiple sources [24]. Both epidemiological field studies [24] and molecular typing [4] provide evidence for the persistence and transmission of infection amongst badgers, which is consistent with their role as a maintenance host. Although increases in their abundance may have contributed to the emergence of wild hosts as significant players in the epidemiology of TB, the risk of transmission to cattle posed by each host species is also related to the number of viable mycobacteria excreted [21,24] and to behavioral, ecological, and farm management factors [6,7,25], which determine rates of direct and indirect contact with wildlife [2]. These factors need to be considered alongside the data presented here on the composition of multiple-host systems in order to assess the relative roles of each species, and so identify and effectively target reservoirs in disease control programmes.

Although epistemic uncertainty was not explicitly incorporated in our model, the dearth of good-quality data on TB prevalence, host abundance, and the sensitivity and specificity of the diagnostic tests contributed to wide ranges for many of the estimates. Surveys employing random sampling are required to generate higher quality prevalence data for domestic and wild hosts throughout Europe. Reliable and contemporary estimates of wild host abundance would also be required [26]. For example, true prevalence in wild boar in the Iberian Peninsula was estimated from the combined results of 10 different surveys (Supplementary Materials Table S8). The confidence interval of the estimate is a function of the variability in prevalence and of the uncertainty in our estimate of prevalence due to heterogeneity between studies [18,27]. Increasing the number of studies included in the model risks increasing the uncertainty of the combined estimate, while considering fewer studies risks failing to incorporate variability in TB prevalence. Similarly, estimates of the performance of the skin tests (single intradermal tuberculin test, SITT, and single comparative intradermal tuberculin test, SCITT) used in cattle were based on numerous studies performed in several European regions (Supplementary Materials Table S1). The sensitivity and specificity of these tests is influenced not only by the performance of the operator and the tuberculin used, but also in relation to individual and farm management factors [28]. The wide range of our estimates likely reflects the variability in the performance of these tests under field conditions.

The numbers of infected non-bovine domestic species in Central–Western Europe and Britain and Ireland may be under-estimated in our study, as they were based solely on slaughterhouse surveillance data. In contrast, in the Iberian Peninsula estimates of TB prevalence in non-bovine domestic animals are likely to be more accurate, being derived from surveys with random sampling and disease control programs (Supplementary Materials Table S8). Nevertheless, the estimates of true TB prevalence in sheep in the Iberian Peninsula vary widely due to the wide uncertainty in apparent prevalence and in the sensitivity of the diagnostic test (Supplementary Materials Tables S1 and S8), due to the poor data availability for this species, which until recently was not considered a maintenance host [16,17].

The assumption of constant TB prevalence, host abundance, and diagnostic test performance within the timeframe of collection of the data used for each region was an undesirable but necessary premise which may not hold in some cases, particularly where control programs led to variations in TB prevalence in cattle [29], or where the abundance of some wild species varied over time [19,20,22]. To minimize this effect, the narrowest possible time range of data was used for each study region. Furthermore, these variations are usually relatively small scale and incremental over the course of a few years and so should largely be accommodated within the wide range of the estimates generated. Instances where we assumed zero prevalence (Supplementary Materials) should be regarded as indicative of the absence of detection rather than an absence of infection.

The role of wildlife and non-bovine domestic animals in the epidemiology of TB in cattle indicates the need to seriously consider the control of infection risks from these populations, particularly where reservoir status [3] can be demonstrated. However, spatial variation in the relative contributions of several host species does suggest that effective disease control will necessitate combinations of different approaches tailored to specific epidemiological situations. For example, test and cull interventions could usefully be extended to epidemiologically significant non-bovine domestic hosts [8,17,30]. Intervention in wildlife populations is more challenging and it is likely that control may require a combination of measures to decrease prevalence, contact with domestic species, and the excretion of viable mycobacteria [31]. Several approaches to these aims are currently being evaluated in the field (reviewed by Gortázar et al. [31]).

Changes in land use [20], game [19], and farming [6,7] practices are likely to have played a role in the emergence of some wildlife populations as important TB hosts in each region, through effects on the abundance, prevalence, and opportunities for transmission. Therefore, it follows that farming and game management systems can be manipulated in order to strengthen biosecurity and decrease direct and indirect contacts between wildlife and cattle, thus reducing transmission [31,32]. Such approaches generally face fewer practical challenges than interventions in wildlife populations [31,32], although further work is required to demonstrate their effectiveness and encourage widespread adoption.

The evidence provided here indicates that in some regions of Europe, TB is a truly multi-host disease within communities comprising cattle and significant populations of non-bovine domestic and wild species. While cattle will remain the key target of TB control due to their economic relevance, control strategies excluding other epidemiologically relevant hosts are unlikely to lead to effective control, whilst complete eradication of the pathogen may be unachievable with the tools presently available. The present quantification of the host community is one of several steps needed to understand the dynamics of transmission within multi-host systems. TB control programs need to address the dynamics of infection in multi-host systems if significant progress is to be achieved.

## 4. Materials and Methods

The European regions included in the study were largely non-OTF Britain and Ireland (Republic of Ireland and United Kingdom), the Iberian Peninsula (mainland Spain and Portugal), and OTF Central-Western Europe (mainland France and Germany). These three regions were chosen as they represent different epidemiological contexts, and because the two non-OTF regions together hold >80% of the infected cattle herds in the European Union [14]. Only species for which there was evidence for TB maintenance host status were considered: cattle, goats, sheep, free-range pigs, farmed cervids (several species), wild boar (*Sus scrofa*), red deer (*Cervus elaphus*), fallow deer (*Dama dama*), and badgers (*Meles meles*) [1,16,17]. A geographically structured analysis of the data was necessary for some country-species combinations, where a wide regional variation in TB prevalence or host abundance had been reported. These cases are highlighted in the Supplementary Materials.

True prevalence was estimated in a Bayesian framework with the package “prevalence” [33] in R 3.3.2 [34]. In all models, the first 10,000 iterations were discarded and the true prevalence derived from the following 100,000 iterations, unless otherwise specified in the Supplementary Materials. The posterior distributions of true prevalence and host population size were multiplied to estimate the TB-infected host population, using package “mc2d” [35], by 100,000 iterations. Uncertainty was not explicitly incorporated in the model, but it is included in the variability [18,33]. Two chains with different initial values were run, and model convergence was assessed by the visual inspection of autocorrelation and traceplots and computation of Brooks–Gelman–Rubin and Geweke tests [36,37,38]. 

The following model was employed:

(1) $$N_{i}=Hp\times \frac{Ap+Sp-1}{Se+Sp-1}$$

where *N_i_* = Number of infected animals, *Hp* = Host population size, *Ap* = Apparent prevalence, *Se* = Sensitivity of the diagnostic test, and *Sp* = Specificity of the diagnostic test. 

A beta (1,1) distribution was used as uninformative prior for true prevalence, while for the sensitivity and specificity of diagnostic tests (Supplementary Materials Table S1), apparent prevalence and host abundance (Supplementary Materials Tables S2–S10), prior information was derived from an extensive literature search using Scopus, Web of Science, and Google Scholar for scientific literature and Google for national and European official data. Combinations of the following keywords were used: “bovine tuberculosis”, “Mycobacterium bovis”, “Mycobacterium caprae”, “Europe”, “France”, “Germany”, “Ireland”, “Portugal”, “Spain”, “United Kingdom”, “cattle”, “goat”, “sheep”, “pig”, “farmed deer”, “wild boar”, “red deer”, “fallow deer”, “badger”, “prevalence”, “sensitivity”, “specificity”, “population”, and “hunting”. Abstracts of the retrieved bibliography were examined to select TB surveys with a random sampling design, data from TB eradication programs, livestock population, and abattoir surveillance data, evaluation of diagnostic tests, estimates of wildlife abundance, hunting statistics, and the proportion of the population hunted (Supplementary Materials). Preference was given to prevalence data obtained in the scope of TB eradication programs employing the single intradermal tuberculin test (SITT) or the single comparative intradermal tuberculin test (SCITT).

Priors derived from meta-analyses of the sensitivity and specificity of TB diagnostic tests and from estimates of wildlife host populations were specified in the model as expert opinion [19]:

(2) $$p\sim PERT\;\left(a,\;b,\;c\right)$$

where *a* = minimum, *b* = most likely and *c* = maximum.

Priors derived from the raw data of sensitivity and specificity of diagnostic tests were specified in the model as beta distribution [18]:

(3) $$p\sim Beta\left(s+1,\;n-s+1\right)$$

where *s* = number of true positives (for sensitivity) and true negatives (for specificity) and *n* = number of animals infected (for sensitivity) and not infected (for specificity). 

The performance of the combination in series of two diagnostic tests, was modeled following the equations [39]:

(4) $$Se_{combined}=Se_{test1}\times Se_{test2}$$

(5) $$Sp_{combined}=Sp_{test1}+Sp_{test2}-\left(Sp_{test1}\times Sp_{test2}\right)$$

When several distributions were available for the same prior (e.g., several TB surveys for the same species and region), they were combined by a probability tree using the package “mc2d” [33]. Proportional weights were assigned to each distribution, as reported in the Supplementary Materials, based on the evaluation of the study design, sample size, geographical scope, and diagnostic methods by two authors. When TB was reported in a given species and country but apparent prevalence data was not available, estimates were extrapolated from the other countries in the same study region, as reported in the Supplementary Materials. When the presence of TB had not been reported for a given species and country, null prevalence was assumed (Supplementary Materials Tables S2,S5,S8).

Domestic host population data were retrieved from official statistical sources [40] and included all animals irrespective of age, sex, or management, except for pigs, where only free-ranging animals were considered at risk of infection. In the Iberian Peninsula, apparent prevalence data for TB in free-ranging pigs is available, so here we considered the free-range pig population [41,42]. In Britain and Ireland and Central–Western Europe, true prevalence in pigs was modeled from abattoir surveillance data, so the whole domestic swine population was considered. 

Wildlife population abundance was modeled as expert opinion Equation (2), when published estimates were available; or else based on official data on the number of animals hunted, corrected by the estimated proportion of the population hunted:

(6) $$Hp=\frac{Hb}{P_{hunt}}$$

where *Hp* = estimated host population size, *Hb* = number of animals hunted in a given year, and *P_hunt_* = estimated proportion of the population hunted annually, based on published data for wild boar [43,44,45,46], and deer [47,48,49]. 

The proportion of infected individuals by species and the ratio of TB-infected non-bovine hosts to cattle were estimated in a Bayesian framework from the posterior distribution of the number of infected animals. Simpson’s index (D) was calculated with the R package “vegan” [50], based on the median of the posterior distribution of the number of infected animals, allowing estimation of the probability that two animals drawn at random from the infected host community belong to the same species [51]. Plots were produced in R with the package “ggplot2” [52] and maps in QGIS 2.18.0 [53].

## Figures and Tables

**Table 1:** Estimates of numbers of tuberculosis (TB)-infected hosts by species and region. Posterior distribution of the number of infected hosts at any given time point reported as median and 95% credible interval.

Host Species		Estimated Number of Infected Hosts							
		Britain and Ireland		Central–Western Europe		Iberian Peninsula		Total	
		Median	Credible Interval_95_	Median	Credible Interval_95_	Median	Credible Interval_95_	Median	Credible Interval_95_
**Cattle**	Cattle	29,871	1279–89,606	2303	98–9460	6175	253–22,356	38,349	1630–121,422
**Non-bovine domestic**	Goat	21	1–83	9	0–36	145,906	106,874–232,240	145,936	106,875–232,359
	Sheep	4	0–20	3	0–16	32.430	1343–146,771	32,437	1343–146,807
	Pigs	7	0–33	3	0–22	25.392	1528–60,716	25,402	1528–60,771
	Cervids (farmed)	73	3–387	26	1–101	21	1–115	120	5–603
**Wildlife**	Wild boar	74	8–325	7118	846–22,716	229,629	103,787–693,486	236,821	104,641–716,527
	Red deer	119	5–452	1912	68–9,033	20,134	953–62,764	22,165	1026–72,249
	Fallow deer	10,621	559–24,259	0	0	10,693	659–35,678	21,314	1218–59,937
	Badger	91,643	62,310–148,225	3231	940–6756	20,403	8543–39,957	115,277	71,793–194,938
Total		132,433	64,165–263,390	14,605	1953–48,140	490,783	223,941–1,294,082	637,821	290,059–1,605,612

**Table 2:** Ratio of estimated TB-infected non-bovine domestic and wild hosts to cattle by region. Ratios calculated based on the posterior distribution of the number of infected hosts.

Region	Class of Host	Ratio Non-Bovine Domestic and Wildlife Hosts/Cattle	
		Median	Credible Interval_95_
**Britain and Ireland**	Non-bovine domestic species	0.004	0.0006–0.03
	Wildlife	3.3	1.3–19.6
	Total	3.3	1.3–19.6
**Central-Western Europe**	Non-bovine domestic species	0.02	0.003–0.2
	Wildlife	6.8	1.4–58.3
	Total	6.8	1.4–58.5
**Iberian Peninsula**	Non-bovine domestic species	35.0	9.2–342
	Wildlife	49.3	11.3–522
	Total	84.3	20.5–864

## Supplementary Materials

The following are available online at https://www.mdpi.com/2076-0817/9/6/421/s1, Table S1. Summary of the prior distributions and supportive references for the sensitivity and specificity of the diagnostic tests, Table S2. Summary of the posterior distributions of the true prevalence by host species in Britain and Ireland and supportive references, Table S3. Summary of the posterior distributions of the abundance by host species in Britain and Ireland and supportive references, Table S4. Summary of the posterior distributions of the number of infected animals by host species in Britain and Ireland, Table S5. Summary of the posterior distributions of the true prevalence by host species in Central–Western Europe and supportive references, Table S6. Summary of the posterior distributions of the abundance by host species in Central–Western Europe and supportive references, Table S7. Summary of the posterior distributions of the number of infected animals by host species in Central–Western Europe, Table S8. Summary of the posterior distributions of the true prevalence by host species in the Iberian Peninsula and supportive references, Table S9. Summary of the posterior distributions of the abundance by host species in the Iberian Peninsula and supportive references, Table S10. Summary of the posterior distributions of the number of infected animals by host species in the Iberian Peninsula.
