# Supplementary material for: Quantification of the Animal Tuberculosis Multi-Host Community Offers Insights for Control
Source: Pathogens. 2020 May 28;9(6):421. doi: 10.3390/pathogens9060421 (PMC7350387; doi:10.3390/pathogens9060421)
Supplement: Supplementary file 1 [file pathogens-09-00421-s001.pdf]

Supplementary Material

Quantification of the animal tuberculosis multi-host community offers insights for control

Nuno Santos, Céline Richomme, Telmo Nunes, Joaquín Vicente, Paulo Célio Alves, José De la Fuente,

Margarida Correia-Neves, María-Laura Boschiroli, Richard Delahay, Christian Gortázar

Table S1. Summary of the prior distributions and supportive references for the sensitivity and specificity of the diagnostic tests.

Table S2. Summary of the posterior distributions of the true prevalence by host species in Britain and Ireland and supportive references.

Table S3. Summary of the posterior distributions of the abundance by host species in Britain and Ireland and supportive references.

Table S4. Summary of the posterior distributions of the number of infected animals by host species in Britain and Ireland.

Table S5. Summary of the posterior distributions of the true prevalence by host species in Central-Western Europe and supportive references.

Table S6. Summary of the posterior distributions of the abundance by host species in Central-Western Europe and supportive references.

Table S7. Summary of the posterior distributions of the number of infected animals by host species in Central-Western Europe.

Table S8. Summary of the posterior distributions of the true prevalence by host species in the Iberian Peninsula and supportive references.

Table S9. Summary of the posterior distributions of the abundance by host species in the Iberian Peninsula and supportive references.

Table S10. Summary of the posterior distributions of the number of infected animals by host species in the Iberian Peninsula.

Host species	Diagnostic test	Sensitivity			Specificity		
		Prior beta distribution (α, β)	Median (CI_{95})	References (proportional weight if combination of priors)	Prior beta distribution (α, β)	Median (CI_{95})	References (proportional weight if combination of priors)
Cattle	SITT	14.69673<br>7.269349	0.674<br>(0.464-0.845)	[28,54-56] - 0.25 weight each reference	18.6233<br>3.290033	0.861<br>(0.677-0.963)	[28,54,56] - 0.33 weight each reference
	SCITT	16.84007<br>12.99837	0.566<br>(0.387-0.734)	[28,56,57] - 0.33	46.7429<br>1.885948	0.967<br>(0.892-0.996)	[28,56,57] - 0.33
Goats	SITT	94<br>39	0.708<br>(0.627-0.781)	[58]	925<br>14	0.985<br>(0.976-0.992)	[59]
	SCITT	12.17448<br>7.704688	0.616<br>(0.395-0.808)	[58,60] - 0.50	938<br>1	0.999<br>(0.996-1)	[59]
	Meat inspection - Bacteriological culture (in series)	9.397363<br>8.010643	0.545<br>(0.311-0.760)	[17,61]	482.424<br>1.48191	0.998<br>(0.990-1)	[17,62]
Sheep	ELISA-Interferon γ assay (in series)	6.114153<br>3.119444	0.674<br>(0.348-0.910)	[17]	10.10635<br>2.068343	0.848<br>(0.585-0.976)	[17]
	Meat inspection - Bacteriological culture (in series)	9.397363<br>8.010643	0.545<br>(0.311-0.760)	[17,61]	482.424<br>1.48191	0.998<br>(0.990-1)	[17,62]
Pigs	ELISA	158.2718<br>30.73391	0.839<br>(0.782-0.886)	[63]	261.2776<br>5.232157	0.982<br>(0.961-0.993)	[63]
	Meat inspection- Bacteriological culture (in series)	4.162443<br>3.188561	0.573<br>(0.226-0.873)	[64]	999.9999<br>2.0000	0.998<br>(0.995-1)	[64]
Wild boar	ELISA	77<br>21	0.788<br>(0.700-0.861)	[65]	105<br>1	0.993<br>(0.966-1)	[65]
	Gross pathology	12.36278<br>3.682683	0.782<br>(0.545-0.934)	[66] - 0.70<br>[64] - 0.30	91<br>1	0.992<br>(0.960-1)	[64]
	Bacteriological culture	19<br>5	0.800<br>(0.611-0.926)	[64]	91<br>1	0.992<br>(0.961-1)	[64]
Cervids	Meat inspection	14.12171<br>8.873023	0.618<br>(0.412-0.797)	[67]	1.740564<br>1.283729	0.594<br>(0.099-0.966)	[67]
	Gross pathology	33.75777<br>8.935112	0.795<br>(0.658-0.897)	[67] - 0.70<br>[68] - 0.30	13.24651<br>1.935955	0.889<br>(0.671-0.984)	[67] - 0.70<br>[68] - 0.30
	Gross pathology – Bacteriological culture (in series)	17.58386<br>12.61998	0.584<br>(0.405-0.749)	[67]	15.88274<br>1.288249	0.941<br>(0.766-0.996)	[67]
	Bacteriological culture	97.74953<br>34.81664	0.739<br>(0.660-0.808)	[67]	4.154772<br>1.087542	0.831<br>(0.397-0.992)	[67]
	ELISA	38<br>37	0.507<br>(0.374-0.639)	[69]	49<br>3	0.936<br>(0.846-0.982)	[69]
	SITT	999.8444<br>215.8183	0.823<br>(0.801-0.843)	[67]	999.9999<br>318.2055	0.759<br>(0.735-0.781)	[67]
Badger	Bacteriological culture post-mortem standard	23.70861<br>19.88225	0.545<br>(0.397-0.687)	[70]	999.9999<br>3.002004	0.997<br>(0.993-0.999)	[71,72] - 0.50
	Bacteriological culture post-mortem detailed	47.88388<br>7.212247	0.874<br>(0.769-0.944)	[73]	999.9999<br>3.002004	0.997<br>(0.993-0.999)	[71,72] - 0.50

SITT – single intradermal tuberculin test

SCITT – single comparative intradermal tuberculin test

ELISA – enzyme-linked immunosorbent assay

Gross pathology – detailed *post-mortem* inspection to detect macroscopic lesions

Meat inspection – standard *post-mortem* inspection to detect macroscopic lesions, performed in slaughterhouse or in the field

Table S1. Summary of the prior distributions and supportive references for the sensitivity and specificity of the diagnostic tests. No data available on the specificity of small ruminant meat inspection – assumed the specificity of cattle slaughterhouse inspection [62]. No data available on the sensitivity and specificity of pig meat inspection – assumed the sensitivity and specificity of gross pathology in wild boar [64]. No data available on the specificity of badger post-mortem bacteriological culture – assumed the specificity of badger ante-mortem bacteriological culture [71,72].

Host species	Regionalization	Country	Diagnostic test (proportional weight if combination of priors)	True prevalence			
				Median	Credible Interval 95	Brooks-Gelman-Rubin test	References
Cattle	Scotland	United Kingdom	SCITT	9.634635e-5	4.157268e-6<br>0.0003184139	1.0001	[74]
	England, Wales, Northern Ireland		SCITT	0.002290942	9.643742e-5<br>0.007447301	1.0181 (1)	[75]
	n.a.	Republic of Ireland	SCITT	0.001286101	5.211055e-5<br>0.004153798	1.0041 (1)	
Goat	n.a.	United Kingdom<br>Republic of Ireland	Meat inspection – Bacteriological culture (in series)	0.000125141	4.879438e-6<br>0.0006586857	1.0002	[75,77]
Sheep	n.a.	United Kingdom<br>Republic of Ireland	Meat inspection – Bacteriological culture (in series)	1.380585e-7	5.198041e-9<br>7.393829e-7	1.0001	
Pig	n.a.	United Kingdom<br>Republic of Ireland	Meat inspection - Bacteriological culture (in series)	9.987239e-7	4.113228e-8<br>5.269530e-6	1.0000	
Cervids (farmed)	n.a.	United Kingdom<br>Republic of Ireland	SITT	0.001560901	5.757917e-5<br>0.008233292	1.0000	[76]
Wild boar	n.a.	United Kingdom	Bacteriological culture	0.03757993	0.004697831<br>0.09193747	1.0002	[78,79]
Red deer	Scotland	United Kingdom	n.a.	0	n.a.	n.a.	[80,81]
	England, Wales, Northern Ireland		Bacteriological culture	0.008246421	0.0003860000<br>0.02949672	1.0001	
	n.a.	Republic of Ireland					
Fallow deer	Scotland	United Kingdom	n.a.	0	n.a.	n.a.	
	England, Wales, Northern Ireland		Bacteriological culture	0.04262090	0.002170639<br>0.09164222	1.0004	
	n.a.	Republic of Ireland					
Badger	Scotland (2)	United Kingdom	n.a.	0	n.a.	n.a.	
	Endemic regions (3)		Bacteriological culture *post-mortem* detailed (0.33)	0.2201824	0.1664002<br>0.2865286	1.0001	[82]
			Bacteriological culture *post-mortem* detailed (0.33)	0.1738025	0.1461464<br>0.2087118	1.0000	[83]
			Bacteriological culture *post-mortem* detailed (0.33)	0.2478713	0.1599084<br>0.3579990	1.0000	[84]
			Combined	0.207	0.151<br>0.327		
	Non-endemic regions (4)		Bacteriological culture *post-mortem* detailed	0.04643781	0.01391974<br>0.1042301	1.0001	[82]
	n.a.	Republic of Ireland	Bacteriological culture *post-mortem* detailed	0.1270293	0.1124526<br>0.1474287	1.0000	[85]

(1) 1,000,000 iterations with 1,000,000 as burnin
(2) Not detected – Null prevalence assumed
(3) Wales, Northern Ireland and the following regions of England: Southwest and West Midlands
(4) The remainder of England
n.a. – not applicable

Table S2. Summary of the posterior distributions of the true prevalence by host species in Britain and Ireland and supporting references. Goat, sheep and pig TB prevalence based on the number of infected animals identified by abattoir surveillance [76,77]. TB prevalence in farmed cervids based on the number animals undergoing intradermal tuberculin tests (test assumed to be SITT) [76]. TB prevalence in badgers in the high-risk areas based on passive surveillance of road traffic accidents in the high-incidence counties of Wales from 2005-2006 [82], in Northern Ireland from 1998-2011 [83] and in the county of Cheshire in England [84]. In the low-risk area from passive surveillance of road traffic accidents in the low-incidence counties of Wales from 2005-2006 [82].

Host species	Regionalization	Country	Host population			
			Exact	Median	Credible Interval $_{95}$	References
Cattle	Scotland	United Kingdom	1,694,411			[86]
	England, Wales, Northern Ireland		8,092,589			
	n.a.	Republic of Ireland	7,363,500			
Goat	n.a.	United Kingdom	105,000			
		Republic of Ireland	9,860 (1)			[77]
Sheep	n.a.	United Kingdom	23,239,000			
		Republic of Ireland	3,981,810			
Pig	n.a.	United Kingdom	4,713,000			
		Republic of Ireland	1,616,360			
Cervids (farmed)	n.a.	United Kingdom	27,000			[67]
		Republic of Ireland	20,000			[87,88]
Wild boar	n.a	United Kingdom		2,000	647 – 5,835	[89,90]
Red deer	Scotland	United Kingdom		439,895	253,107 – 686,560	[89-92]
	England, Wales, Northern Ireland			11,279	6,490 – 17,604	
	n.a.	Republic of Ireland		3,009	1,832 – 4,626	[92]
Fallow deer	Scotland	United Kingdom		19,536	14,356 – 25,382	[90,91]
	England, Wales, Northern Ireland			244,464	179,644 – 317,618	
	n.a.	Republic of Ireland		8,374	5,108 – 12,804	[22,91-93]
Badger	Scotland	United Kingdom		42,820	26,280 – 59,360	[96,97]
	Endemic regions			354,259	304,642 – 404,198	[22,94-95]
	Non-endemic regions			164,900	132,940 – 197,540	[22,95]
	n.a.	Republic of Ireland		83,832	75,597 – 91,866	[98]

(1) 2015 estimate

n.a. – not applicable

Table S3. Summary of the posterior distributions of the abundance by host species in Britain and Ireland and supporting references. Deer population in Scotland: estimated deer population in the UK [89,90] multiplied by the proportion of the UK deer population in Scotland [91]. Badger population in the high-risk area of the UK: badger population in Northern Ireland [94] + badger population in England and Wales [94], multiplied by the proportion of setts in high-risk regions [96]. Badger population in the low-risk area of the UK: badger population in England and Wales [94], multiplied by the proportion of setts in low-risk regions [96]. Badger population in Scotland: number of setts in Scotland [97] multiplied by the average size of badger social groups in Scotland [98].

Host species	Regionalization	Country	Infected hosts		
			Median	Credible Interval 95	Geweke test (p)
Cattle	Scotland	United Kingdom	162	7 – 540	-0.448 (0.654)
	England, Wales, Northern Ireland		20,480	905 – 59,286	0.342 (0.733)
	n.a.	Republic of Ireland	9,229	367 – 29,780	0.181 (0.856)
		Total	29,871	1,279 – 89,606	
Goat	n.a.	United Kingdom	19	1 – 76	-1.927 (0.054)
		Republic of Ireland	2	0 – 7	-1.927 (0.054)
		Total	21	1 - 83	
Sheep	n.a.	United Kingdom	3	0 – 17	1.159 (0.246)
		Republic of Ireland	1	0 – 3	1.159 (0.246)
		Total	4	0 – 20	
Pig	n.a.	United Kingdom	5	0 – 25	-1.211 (0.226)
		Republic of Ireland	2	0 - 8	-1.211 (0.226)
		Total	7	0 - 33	
Cervids (farmed)	n.a.	United Kingdom	42	2 - 222	-0.433 (0.665)
		Republic of Ireland	31	1 - 165	-0.433 (0.665)
		Total	73	3 - 387	
Wild boar	n.a.	United Kingdom	74	8 - 325	1.825 (0.068)
Red deer	Scotland	United Kingdom	0	n.a.	
	England, Wales, Northern Ireland		94	4 – 354	0.869 (0.385)
	n.a.	Republic of Ireland	25	1 - 98	0.556 (0.579)
		Total	119	5 – 452	
Fallow deer	Scotland	United Kingdom	0	n.a.	
	England, Wales, Northern Ireland		10,283	541 – 23,354	-1.222 (0.222)
	n.a.	Republic of Ireland	338	18 - 905	-1.169 (0.243)
		Total	10,621	559 – 24,259	
Badger	Scotland	United Kingdom	0	n.a.	
	Endemic regions		73,350	50,978 – 118,075	-0.420 (0.675)
	Non-endemic regions		7,648	2,276 – 17,459	0.303 (0.762)
	n.a.	Republic of Ireland	10,645	9,056 – 12,691	-1.206 (0.228)
		Total	91,643	62,310 – 148,225	

n.a. – not applicable

Table S4. Summary of the posterior distributions of the number of infected animals by host species in Britain and Ireland.

Host species	Regionalization	Country	Diagnostic test (proportional weight if combination of priors)	True prevalence			
				Median	Credible Interval 95	Brooks-Gelman-Rubin test	References
Cattle	*Départments* undergoing annual or biennial SITT (1)	France	SITT	0.0004141504	1.670802e-5<br>0.001541546	1.0006	[99]
	Départments undergoing annual or biennial SCITT (2)		SCITT	0.0005631272	2.475756e-5<br>0.001695942	1.0001	
	Départments not undergoing annual or biennial skin test (3)		SCITT	6.629754e-5	2.791990e-6<br>0.0002783877	1.0000	[100]
	n.a.	Germany	SCITT				
Goat	n.a.	France<br>Germany	Meat inspection – Bacteriological culture (in series)	6,79e-6	2.98e-7<br>2.65e-5	1.0000	[77,101]
Sheep	n.a.	France<br>Germany	Meat inspection – Bacteriological culture (in series)	3.231076e-7	1.18e-8<br>1.89e-6	1.0001	[77]
Pig	n.a.	France<br>Germany	Meat inspection - Bacteriological culture (in series)	8.232490e-8	2.959828e-9<br>5.577189e-7	1.0001	[77,102]
Cervids (farmed)	n.a.	France<br>Germany	Bacteriological culture	0.0006950049	3.124447e-5<br>0.002699539	1.0001	[103-105]
Wild boar	Endemic regions (4)	France	Bacteriological culture	0.02337694	0.002798085<br>0.03896244	1.0011	[106]
	Non-endemic regions (5)		n.a.	0 (6)	n.a.	n.a.	
	n.a.	Germany	n.a.	0 (6)	n.a.	n.a.	
Red deer	Endemic regions (4)	France	Bacteriological culture	0.004955674	0.0002343514<br>0.01655158	1.0000	[106]
	Non-endemic regions (5)		n.a.	0 (6)	n.a.	n.a.	
	n.a.	Germany	Bacteriological culture (0.50)	0.009977578	0.0004703266<br>0.03483379	1.0000	[107]
			Bacteriological culture (0.50)	0.004734339	1.952244e-7<br>0.02077311	1.0001	[108]
			Combined	0.00696	0.000241<br>0.0316		
Fallow deer	n.a.	France<br>Germany	n.a.	0 (6)	n.a.	n.a.	
Badger	Endemic regions (4)	France	Bacteriological culture *post-mortem* standard protocol	0.07561729	0.05360701<br>0.1107847	1.0000	[106]
	Non-endemic regions (5)		n.a.	0 (6)	n.a.	n.a.	
	n.a.	Germany	n.a.	0 (6)	n.a.	n.a.	

(1) *Départments* undergoing annual or biennial testing by SITT in cattle: Alpes-Maritimes, Bouches-du-Rhône, Landes, Lot-et-Garonne, Savoie, Var

(2) *Départments* undergoing annual or biennial testing by SCITT in cattle: Cote d’Or

(3) *Départments* not undergoing annual or biennial testing by skin test in cattle: all others in France

(4) *Départments* containing at-risk areas [107]: Ardennes, Ariège, Charente, Charente-Maritime, Corrèze, Côte-d'Or, Dordogne, Haute-Garonne, Gironde, Loir-et-Cher, Lot, Lot-et-Garonne, Marne, Pyrénées-Atlantiques, Seine-Maritime, Haute-Vienne

(5) *Départments* without at-risk areas [107]: all others in France

(6) Not detected – Null prevalence assumed

n.a. – not applicable

Table S5. Summary of the posterior distributions of the true prevalence by host species in Central-Western Europe and supporting references. TB prevalence in goats and pigs estimated from abattoir surveillance data [102,103]. TB prevalence in sheep estimated assuming no detections from slaughterhouse surveillance [102,103]. Estimates of TB prevalence in wild boar, red deer and badgers in endemic regions based on targeted surveillance in level 3 *départments* from 2011-2014 [107].

Host species	Regionalization	Country	Host population			
			Exact	Median	Credible Interval $_{95}$	References
Cattle	*Départments* undergoing annual or biennial SITT testing	France	218,800			[77,109]
	Départments undergoing annual or biennial SCITT testing		234,900			
	Départments not undergoing annual or biennial skin testing		18,979,020			
	n.a.	Germany	12,365,500			
Goat	n.a.	France	1,213,000			[77]
		Germany	140,000			
Sheep	n.a.	France	6,877,000			
		Germany	1,579,790			
Pig	n.a.	France	13,353,000			
		Germany	27,577,570			
Cervids (farmed)	n.a.	France	30,000			[110]
		Germany	7,500			[66]
Wild boar	Endemic regions	France		294,632	212,739 – 851,727	Hunting bag: [111,112] Proportion hunted: [43-46]
	Non-endemic regions	France		1,231,896	889,492 – 3,561,190	
	n.a.	Germany		1,269,034	916,308 – 3,668,550	
Red deer	Endemic regions	France		44,952	30,546 – 68,456	Hunting bag: [111,1123] Proportion hunted: [47-49] Expert opinion: [113-114]
	Non-endemic regions	France		141,163	95,751 – 214,245	
	n.a.	Germany		244,375	167,762 – 368,331	
Fallow deer		France		3,966	2,409 – 6,493	
	n.a.	Germany		233,925	142,092 – 382,974	
Badger	Endemic regions	France		43,058	3,818 – 78,827	[115,116]
	Non-endemic regions			200,866	16,069 – 482,078	
	n.a.	Germany		225,468	84,407 – 546,131	[117]

n.a. – not applicable

Table S6. Summary of the posterior distributions of the abundance by host species in Central-Western Europe and supporting references. Red deer and fallow deer populations estimated by combination of expert opinion (0.30 weight) [114,115]; and number of animals hunted (average 2015-2016 hunting seasons) [112,113], multiplied by the proportion of the population hunted annually (0.70 weight) [34-36]. Badger population in France estimated based on reported range of densities [116,117], multiplied by the proportion of French territory in endemic/non-endemic *départments* [107].

Host species	Regionalization	Country	Infected hosts		
			Median	Credible Interval 95	Geweke test (p)
Cattle	*Départments* undergoing annual or biennial SITT testing	France	92	4 - 340	-0.200 (0.842)
	Départments undergoing annual or biennial SCITT testing		133	6 - 398	-0.794 (0.427)
	Départments not undergoing annual or biennial skin testing		1,258	53 – 5,281	-0.064 (0.949)
	n.a.	Germany	820	35 – 3,441	-0.064 (0.949)
		Total	2,303	98 – 9,460	
Goat	n.a.	France	8	0 - 32	1.215 (0.224)
		Germany	1	0 - 4	1.215 (0.224)
		Total	9	0 - 36	
Sheep	n.a.	France	2	0 - 13	0.825 (0.410)
		Germany	1	0 - 3	0.825 (0.410)
		Total	3	0 - 16	
Pig	n.a.	France	1	0 - 7	-0.468 (0.640)
		Germany	2	0 - 15	-0.468 (0.640)
		Total	3	0 - 22	
Cervids (farmed)	n.a.	France	21	1 - 82	-1.635 (0.102)
		Germany	5	0 - 20	-1.635 (0.102)
		Total	26	1 - 102	
Wild boar	Endemic regions	France	7,118	846 – 22,716	-0.791 (0.429)
	Non-endemic regions		0	n.a.	
	n.a.	Germany	0	n.a.	
		Total	7,118	846 – 22,716	
Red deer	Endemic regions	France	220	10 – 809	0.130 (0.897)
	Non-endemic regions		0	n.a.	
	n.a.	Germany	1,692	58 – 8,224	1.071 (0.284)
		Total	1,912	68 – 9,033	
Fallow deer	n.a.	France	0	n.a.	
		Germany	0	n.a.	
		Total	0	n.a.	
Badger	Endemic regions	France	3,231	940 – 6,756	-0.408 (0.627)
	Non-endemic regions		0	n.a.	
	n.a.	Germany	0	n.a.	
		Total	3,231	940 – 6,756	

n.a. – not applicable

Table S7. Summary of the posterior distributions of the number of infected animals by host species in Central-Western Europe.

Host species	Regionalization	Country	Diagnostic test (proportional weight if combination of priors)	True prevalence			
				Median	Credible Interval $_{95}$	Brooks-Gelman-Rubin test	References
Cattle	n.a.	Spain	SITT	0.000861650	3.470549e-5 0.003217358	1.0060 (1)	[75]
		Portugal	SCITT	0.000286265	1.236615e-5 0.0008420191	1.0004	
Goat	No long-term eradication program in caprines (2)	Spain Portugal	SCITT	0.05136874	0.03789387 0.08299825	1.0006 (1)	[118]
	Long term eradication program in caprine (3)	Spain	SITT	0.000443180	1.697337e-50.001985997	1.0001	[119]
Sheep	n.a.	Spain Portugal	ELISA-Interferon γ assay (in series)	0.00179478	0.0000735947 0.008128424	1.0000	IREC (unpub.)
Pig (free-range)	n.a.	Spain Portugal	ELISA	0.008186200	0.0005520730 0.02203972	1.0001	[48]
Cervids (farmed)	n.a.	Spain	SITT	0.00239126	9.142929e-5 0.0122240	1.0003	[120]
Wild boar	Endemic regions (4)	Spain	Gross pathology (0.25)	0.7609085	0.6300402 0.9670142	1.0007	[121]
			Gross pathology (0.25)	0.5465099	0.4205817 0.8054564	1.0002	[122]
			ELISA (0.25)	0.7031711	0.5404390 0.9510652	1.0004	[123]
			ELISA (0.25)	0.4348425	0.3147407 0.6702440	1.0001	[124]
			Combined	0.630	0.351 0.939		
		Portugal	ELISA (0.33)	0.2212445	0.1271466 0.3382338	1.0000	[125]
			Bacteriological culture (0.33)	0.2619104	0.1935771 0.3683385	1.0000	
			Meat inspection (0.33)	0.1803245	0.1333477 0.2699077	1.0002	[126]
			Combined	0.222	0.137 0.345		
	Non-endemic regions (5)	Portugal	ELISA	0.001490437	5.44e-5 0.00785	1.0000	[125]
		Spain	Bacteriological culture (0.50)	0.04488553	0.01107392 0.06554483	1.0006	[119]
			Bacteriological culture (0.50)	0.02458174	0.003057920 0.04386654	1.0000	[127]
			Combined	0.0333	0.0048 0.0612		
Red deer	Endemic regions (4)	Spain	Bacteriological culture (0.50)	0.04295531	0.002312960 0.1101596	1.0012	[121]
			ELISA (0.50)	0.03272815	0.001638379 0.09346738	1.0000	[122]
			Combined	0.0383	0.00188 0.106		
	Non-endemic regions (5)		Bacteriological culture	0.01544085	0.0007688408 0.03533709	1.0004	[119]

	n.a.	Portugal	Bacteriological culture (0.50)	0.1315712	0.007944894 0.2575010	1.0000	[125]
			Meat inspection (0.50)	0.05226237	0.002557873 0.1347850	1.0006	[126]
			Combined	0.0773	0.00359 0.241		
Fallow deer	Endemic regions (4)	Spain	Gross pathology - Bacteriological culture (in series) (0.33)	0.2369761	0.03059517 0.4489423	1.0002	[128]
			Gross pathology (0.33)	0.1329399	0.009332847 0.3075522	1.0002	[122]
			ELISA (0.33)	0.07530128	0.004632357 0.2074204	1.0001	[123]
			Combined	0.134	0.00866 0.386		
	Non-endemic regions (5)		Bacteriological culture	0.06292065	0.003367532 0.1592813	1.0001	[119]
	n.a.	Portugal	n.a.	0 (6)	n.a.	n.a.	n.a.
Badger	Euro-Siberian bioregion	Spain Portugal	Bacteriological culture *post-mortem* detailed	0.1608726	0.09463825 0.2488233	1.0000	[129]
	Mediterranean bioregion		n.a.	0 (6)	n.a.	n.a.	

(1) 1,000,000 iterations with 1,000,000 as burnin

(2) Spain: Castilla y León, Murcia

(3) The rest of the Iberian Peninsula

(4) Portugal: As defined in "*Edital nº 1 – Tuberculose em Caça Maior*"; Spain: Andalucia, Castilla la Mancha, Extremadura, Madrid

(5) The remainder of the Iberian Peninsula

(6) Not detected – Null prevalence assumed

n.a. – not applicable

Table S8. Summary of the posterior distributions of the true prevalence by host species in the Iberian Peninsula and supporting references.

Host species	Regionalization	Country	Host population			
			Exact	Median	Credible Interval $_{95}$	References
Cattle	n.a.	Spain	6,588,110			[77]
		Portugal	1,722,160			
Goat	Long term eradication program in caprines	Spain	581,672			[77,130]
	No long-term eradication program in caprines		2,479,758			
		Portugal	339,650			
Sheep	n.a.	Spain	15,963,110			[77]
		Portugal	2,224,690			
Pig (free-range)	n.a.	Spain	2,175,022			[41]
		Portugal	577,825			[42]
Cervids (farmed)	n.a.	Spain		8,873	5,108 – 11,181	[132]; expert opinion [CG, NS]
Wild boar	Endemic regions	Spain Portugal		318,822	230,206 – 921,656	Hunting bag: [132,133] Proportion hunted: [43-46]
				18,484	13,346 – 53,433	
	Non-endemic regions			437,495	315,894 – 1,264,720	
				63,244	45,666 – 182,828	
Red deer	Endemic regions	Spain		490,663	327,189 – 755,986	Hunting bag: [132,133] Proportion hunted: [47-49] Expert opinion: [134-135]
	Non-endemic regions			55,739	37,096 – 85,998	
	n.a.	Portugal		13,399	8,558 – 18,567	
Fallow deer	Endemic regions	Spain		77,738	47,220 – 127,270	
	Non-endemic regions			7,012	2,919 – 11,479	
	n.a.	Portugal		2,718	2,004 – 3,876	
Badger	Euro-Siberian bioregion	Spain Portugal		130,241	62,020 – 203,138	[136-139]
	Mediterranean bioregion			251,719	194,468 – 308,453	[136,137,139]

n.a. – not applicable

Table S9. Summary of the posterior distributions of the abundance by host species in the Iberian Peninsula and supporting references. Red and fallow deer population estimated by combination of expert opinion (0.30 weight) [135,136] and hunting bag [133,134] multiplied for the proportion of the population hunted [34-36] (0.70 weight). Badger population estimated separately for the Euro-Siberian and Mediterranean bioregions of the Iberian Peninsula [140], as very different densities have been reported for each [137-139].

Host species	Regionalization	Country	Infected hosts		
			Median	Credible Interval 95	Geweke test (p)
Cattle	n.a.	Spain	5,685	232 – 20,923	-0.301 (0.763)
		Portugal	490	21 – 1,433	1.360 (0.174)
		Total	6,1775	253 – 22,356	
Goat	Long term eradication program in caprines	Spain	258	10 – 1,170	-0.884 (0.377)
	No long-term eradication program in caprines		128,102	93,990 – 203,233	-0.002 (0.998)
		Portugal	17,546	12,874 – 27,837	-0.002 (0.998)
	n.a.	Total	145,906	106,874 – 232,240	
Sheep	n.a.	Spain	28,463	1,179 – 128,818	0.230 (0.818)
		Portugal	3,967	164 – 17,953	0.230 (0.818)
		Total	32,430	1,343 – 146,771	
Pig (free-range)	n.a.	Spain	20,062	1,207 – 47,972	0.8645 (0.387)
		Portugal	5,330	321 – 12,744	0.8645 (0.387)
		Total	25,392	1,528 – 60,716	
Cervids (farmed)	n.a.	Spain	21	1 - 115	-1.556 (0.120)
		Total	21	1 - 115	
Wild boar	Endemic regions	Spain Portugal	209,445	99,438 – 628,371	-0.837 (0.403)
			4,284	2,057 – 12,960	-0.807 (0.420)
	Non-endemic regions		15,796	2,288 – 51,443	0.669 (0.503)
			104	4 - 712	0.790 (0.429)
	n.a.	Total	229,629	103,787 – 693,486	
Red deer	Endemic regions	Spain	18,259	866 – 57,047	-1.460 (0.144)
	Non-endemic regions		840	40 – 2,246	0.495 (0.621)
	n.a.	Portugal	1,035	47 – 3,471	0.448 (0.654)
		Total	20,134	953 – 62,764	
Fallow deer	Endemic regions	Spain	10,265	636 – 34,392	-0.522 (0.602)
	Non-endemic regions		428	23 – 1,286	-2.296 (0.022)
	n.a.	Portugal	0	n.a.	
		Total	10,693	659 – 35,678	
Badger	Euro-Siberian bioregion	Spain	20,403	8,543 – 39,957	-0.976 (0.329)
	Mediterranean bioregion	Portugal	0	n.a.	
	n.a.	Total	20,403	8,543 – 39,957	

n.a. – not applicable

Table S10. Summary of the posterior distributions of the number of infected animals by host species in the Iberian Peninsula.

References

54. Álvarez, J.; Pérez, A.; Bezos, J.; Marqués, S.; Grau, A.; Sáez, J.L.; Mínguez, O.; de Juan, L.; Domínguez, L. Evaluation of the sensitivity and specificity of bovine tuberculosis diagnostic tests in naturally infected cattle herds using a Bayesian approach. Vet. Microbiol. 2012, 155, 38–43.
55. Casal, C.; Infantes, J.A.; Risalde, M.A.; Díez-Guerrier, A.; Domínguez, M.; Moreno, I.; de Juana, L.; Sáez, J.L.; Juste, R.; Gortázar, C.; et al. Antibody detection tests improve the sensitivity of tuberculosis diagnosis in cattle. Res. Vet. Sci. 2017, 112, 214–221.
56. Pucken, V.B.; Knubben-Schweizer, G.; Döpfer, D.; Groll, A.; Hafner-Marx, A.; Hörmansdorfer, S.; Sauter-Louis, C.; Straubinger, R.K.; Zimmermann, P.; Hartnack, S. Evaluating diagnostic tests for bovine tuberculosis in the southern part of Germany: A latent class analysis. PLoS One 2017, 12, e0179847.
57. Lahuerta-Marin, A.; Milne, M.G.; McNair, J.; Skuce, R.A.; McBride, S.H.; Menzies, F.D.; McDowell, S.J.W.; Byrne, A.W.; Handel, I.G.; Bronsvoort, B.D.C. Bayesian latent class estimation of sensitivity and specificity parameters of diagnostic tests for bovine tuberculosis in chronically infected herds in Northern Ireland. Vet. J. 2018, 238, 15–21.
58. Álvarez, J.; de Juan, L.; Bezos, J.; Romero, B.; Sáez, J.L.; Gordejo, F.R.; Briones, V.; Moreno, M.A.; Mateos, A.; Domínguez, L.; et al. Interference of paratuberculosis with the diagnosis of tuberculosis in a goat flock with a natural mixed infection. Vet. Microbiol. 2008, 128, 72–80.
59. Bezos, J.; Álvarez, J.; Mínguez, O.; Marqués, S.; Martín, O.; Vigo, V.; Pieltain, C.; Romero, B.; Rodríguez, S.; Casal, C.; et al. Evaluation of specificity of tuberculosis diagnostic assays in caprine flocks under different epidemiological situations. Res. Vet. Sci. 2012, 93, 636–640.
60. Gutiérrez, M.; Tellechea, J.; Marín, J.F.G. Evaluation of cellular and serological diagnostic tests for the detection of Mycobacterium bovis-infected goats. Vet. Microbiol. 1998, 62, 281–290.
61. Hardstaff, J.; Nigsch, A.; Dadios, N.; Stärk, K.; Alonso, S.; Lindberg, A. Contribution of meat inspection to animal health surveillance in sheep and goats. EFSA Sup. Pub. 2012, 9, 320E.
62. Bermingham, M.L.; Handel, I.G.; Glass, E.J.; Woolliams, J.A.; Bronsvoort, B.M.C.; McBride, S.H.; Skuce, R.A.; Allen, A.R.; McDowell, S.W.J.; Bishop, S.C. Hui and Walter's latent-class model extended to estimate diagnostic test properties from surveillance data: A latent model for latent data. Sci. Rep. 2015, 5, 11861.
63. Thomas, J.; Infantes-Lorenzo, J.A.; Moreno, I.; Cano-Terriza, D.; de Juan, L.; García-Bocanegra, I.; Domínguez, L.; Domínguez, M.; Gortázar, C.; Risalde, M.A. Validation of a new serological assay for the identification of Mycobacterium tuberculosis complex-specific antibodies in pigs and wild boar. Prev. Vet. Med. 2018, 162, 11–17.
64. Santos, N.; Geraldes, M.; Afonso, A.; Almeida, V.; Correia-Neves, M. Diagnosis of tuberculosis in the wild boar (Sus scrofa): A comparison of methods applicable to hunter-harvested animals. PLoS One 2010, 5, e12663.
65. Boadella, M.; Lyashchenko, K.; Greenwald, R.; Esfandiari, J.; Jaroso, R.; Carta, T.; Garrido, J.M.; Vicente, J.; de la Fuente, J.; Gortázar, C. Serologic tests for detecting antibodies against Mycobacterium bovis and Mycobacterium avium subspecies paratuberculosis in Eurasian wild boar (Sus scrofa scrofa). J. Vet. Diag. Invest. 2011, 23, 77–83.
66. Martín-Hernando, M.P.; Höfle, U.; Vicente, J.; Ruiz-Fons, F.; Vidal, D.; Barral, M.; Garrido, J.M.; de la Fuente, J.; Gortázar, C. Lesions associated with Mycobacterium tuberculosis complex infection in the European wild boar. Tuberculosis 2007, 87, 360–367.
67. European Food Safety Authority. Scientific opinion of the panel on animal health and animal welfare on a request from the European Commission on tuberculosis testing in deer. EFSA J. 2008, 645, 1–34.
68. Martín-Hernando, M.P.; Torres, M.J.; Aznar, J.; Negro, J.J.; Gandía, A.; Gortázar, C. Distribution of lesions in red and fallow deer naturally infected with Mycobacterium bovis. J. Comp. Pathol. 2010, 142, 43–50.
69. Boadella, M.; Barasona, J.A.; Díaz-Sanchez, S.; Lyashchenko, K.P.; Greenwald, R.; Esfandiari, J.; Gortázar, C. Performance of immunochromatographic and ELISA tests for detecting fallow deer infected with Mycobacterium bovis. Prev. Vet. Med. 2012, 104, 160–164.
70. Crawshaw, T.R.; Griffiths, I.B.; Clifton-Hadley, R.S. Comparison of a standard and a detailed postmortem protocol for detecting Mycobacterium bovis in badgers. Vet. Rec. 2008, 163, 473–477.
71. Drewe, J.A.; Tomlinson, A.J.; Walker, N.J.; Delahay, R.J. Diagnostic accuracy and optimal use of three tests for tuberculosis in live badgers. PLoS One 2010, 5, e11196.

72. Buzdugan, S.N.; Chambers, M.A.; Delahay, R.J.; Drewe, J.A. Diagnosis of tuberculosis in groups of badgers: An exploration of the impact of trapping efficiency, infection prevalence and the use of multiple tests. Epidemiol. Infect. 2016, 144, 1717–1727.
73. Corner, L.A.; O'Meara, D.; Costello, E.; Lesellier, S.; Gormley, E. The distribution of Mycobacterium bovis infection in naturally infected badgers. Vet. J. 2012, 194, 166–172.
74. American Pharmacists Association. Bovine tuberculosis: Infection status in cattle in GB, annual surveillance report for the period January to December 2017. In Animal and Plant Health Agency, Report Project SB4500; 2018. Available online: https://assets.publishing.service.gov.uk (accessed on 12 December 2018).
75. DG Sanco. Approved Veterinary Programmes, 2017 Data. Available online: https://ec.europa.eu/food/funding/animal-health/national-veterinary-programmes_en (accessed on 18 May 2020).
76. DEFRA. Statistics on Bovine TB in Non-Bovine Species, 2012–2016 Data. Available online: www.gov.uk/government/statistical-data-sets/other-tb-statistics (accessed on 15 December 2017).
77. Eurostat. Livestock and Meat Production Database, 2017 Data. Available online: http://ec.europa.eu/eurostat/web/agriculture/ data/database (accessed on 18 May 2020).
78. Barlow, A. Living with feral wild boar in the Forest of Dean—Disease risks and contingency plan. APHA Wildlife & Pig Species Expert Groups, APHA Starcross Veterinary Investigation Centre. In Workshop 'Living with Feral Wild Boar; Forestry Commission: 2017. Available online: http://adlib.everysite.co.uk/resources/000/250/259/feralwildboar.pdf (accessed on 12 December 2018).
79. Williamson, S.; Smith, R.; Barlow, A. Surveillance for non-statutory pathogens in culled wild boar in the forest of Dean. In BPEX Project RDOR1035; AHVLA Pig and Wildlife Expert Groups: 2014. Available online: http://apha.defra.gov.uk/documents/surveillance/diseases/wild-boar-surveillance-1516.pdf (accessed on 12 December 2018).
80. Delahay, R.J.; Smith, G.C.; Barlow, A.M.; Walker, N.; Harris, A.; Clifton-Hadley, R.S.; Cheeseman, C.L. Bovine tuberculosis infection in wild mammals in the South-West region of England: A survey of prevalence and a semi-quantitative assessment of the relative risks to cattle. Vet. J. 2007, 173, 287–301.
81. Patterson, A. Report of the 2006–2007 South-west England and Cotswolds survey of tuberculosis in deer. In Tuberculosis Programme; Defra: 2008. Available online: http://defra.gov.uk/animalh/tb (accessed on 15 december 2017).
82. Goodchild, A.V.; Watkins, G.H.; Sayers, A.R.; Jones, J.R.; Clifton-Hadley, R.S. Geographical association between the genotype of bovine tuberculosis in found dead badgers and in cattle herds. Vet. Rec. 2012, 170, 259.
83. Courcier, E.A.; Menzies, F.D.; Strain, S.A.; Skuce, R.A.; Robinson, P.A.; Patterson, I.A.; McBride, K.R.; McCormick, C.M.; Walton, E.; McDowell, S.W.J.; et al. Monitoring Mycobacterium bovis in Eurasian badgers (Meles meles) killed by vehicles in Northern Ireland between 1998 and 2011. Vet. Rec. 2017, 182, 259.
84. Barron, E.S.; Swift, B.; Chantrey, J.; Christley, R.; Gardner, R.; Jewell, C.; McGrath, I.; Mitchell, A.; O'Cathail, C.; Prosser, A.; et al. A study of tuberculosis in road traffic-killed badgers on the edge of the British bovine TB epidemic area. Sci. Rep. 2018, 8, 17206.
85. Byrne, A.W.; Kenny, K.; Fogarty, U.; O'Keeffe, J.J.; More, S.J.; McGrath, G.; Teeling, M.; Martin, S.W.; Dohoo, I.R. Spatial and temporal analyses of metrics of tuberculosis infection in badgers (Meles meles) from the Republic of Ireland: Trends in apparent prevalence. Prev. Vet. Med. 2015, 122, 345–354.
86. Scottish Government (2020) Scottish Agricultural Survey: December 2017. Available online: https://www.gov.scot/publications/results-2017-december-agricultural-survey/ (accessed on 18 May 2020).
87. Quigley, F.C.; Costello, E.; Flynn, O.; Gogarty, A.; McGuirk, J.; Murphy, A.; Egan, J. Isolation of mycobacteria from lymph node lesions in deer. Vet. Rec. 1997, 141, 516.
88. Partridge, T.; Toolan, D.; Egan, J.; More, S. Control of Mycobacterium bovis infection in two sika deer herds in Ireland. Irish Vet. J. 2008, 61, 27.
89. Croft, S.; Chauvenet, A.L.; Smith, G.C. A systematic approach to estimate the distribution and total abundance of British mammals. PLoS One 2017, 12, e0176339.
90. Mathews, F.; Kubasiewicz, L.M.; Gurnell, J.; Harrower, C.A.; McDonald, R.A.; Shore, R.F. A Review of the Population and Conservation Status of British Mammals: Technical Summary; Natural England: Peterborough, UK, 2018.

91. Battersby, J.; Tracking Mammals Partnership. UK mammals: Species status and population trends. In First Report by the Tracking Mammals Partnership, JNCC/Tracking Mammals Partnership, Peterborough: 2005. Available online: http://archive.jncc.gov.uk/pdf/pub05_ukmammals_speciesstatusText_final.pdf (accessed on 12 December 2018).
92. Putman, R.J. Ungulates and their management in Great Britain and Ireland. In European Ungulates and Their Management in the 21st Century; Apollonio, M., Andersen, R., Putman, R., Eds.; Cambridge University Press: Cambridge, UK, 2010.
93. Ward, A.; Graham, C.; Etherigton, T.R.; Delahay, R.J. Estimating the risk of cattle exposure to tuberculosis posed by wild deer relative to badgers in England and Wales. J. Wildl. Dis. 2009, 45, 1104–1120.
94. Reid, N.; Etherington, T.R.; Wilson, G.J.; Montgomery, W.I.; McDonald, R.A. Monitoring and population estimation of the European badger Meles meles in Northern Ireland. Wildl. Biol. 2012, 18, 46–57.
95. Wilson, G.; Harris, S.; McLaren, G. Changes in the British badger population, 1988 to 1997. People's Trust for Endangered Species: London, UK, 1997.
96. Rainey, E.; Butler, A.; Bierman, S.; Roberts, A.M.I. Scottish Badger Distribution Survey 2006–2009. In Estimating the Distribution and Density of Badger Main Setts in Scotland, Report by Scottish Badgers and Biomathematics and Statistics Scotland: 2009. Available online: https://www.scottishbadgers.org.uk/userfiles/file/Main_folder1/C204587-Scottish-Badger-Distribution-Survey-06-09-Results-16-November-2009.pdf (accessed on 12 December 2018).
97. Kruuk, H.H.; Parish, T. Changes in the size of groups and ranges of the European badger (Meles meles L.) in an area in Scotland. J. Anim. Ecol. 1987, 56, 351–364.
98. Sleeman, D.P.; Davenport, J.; More, S.J.; Clegg, T.A.; Collins, J.D.; Martin, S.W.; Williams, D.H.; Griffin, J.M.; O'Boyle, I. How many Eurasian badgers Meles meles L. are there in the Republic of Ireland? Eur. J. Wildl. Res. 2009, 55, 333–344.
99. Cavalerie, L.; Courcoul, A.; Boschiroli, M.L.; Réveillaud, E.; Gay, P. Bovine tuberculosis in France in 2014: A stable situation. Bull. Epidémiol. Anim. Health Nutr. 2015, 71, 4–11.
100. Menge, C.; Köhler, H.; Moser, I.; Conraths, F.J.; Homeier, T. Nationwide cross-sectional study on bovine tuberculosis by intra vitam testing in Germany, 2013–2014. Transbound. Emerg. Dis. 2017, 64, 1236–1242.
101. Agence Française de Sécurité Sanitaire des Aliments. Avis de l'Agence Française de Sécurité Sanitaire des Aliments Relatif au Projet d'Arrêté Modifiant l'arrêté du 15 Septembre 2003 Fixant les Mesures Techniques et Administratives Relatives à la Prophylaxie Collective et à la Police Sanitaire de la Tuberculose des Bovinés et des Caprins; Saisine n. 2009-SA-0300; Agence Française de Sécurité Sanitaire des Aliments; Maisons-Alfort, France, 2010.
102. Agence Française de Sécurité Sanitaire des Aliments. Avis de l'Agence Française de Sécurité Sanitaire des Aliments Relatif à l'évaluation du Risque lié à la Consommation de Viandes Porcines Contaminées par Mycobacterium spp. et en Particulier par Mycobacterium Avium et l'adaptation du Système d'Inspection en Abattoir; Saisine n. 2009-SA-0300; Agence Française de Sécurité Sanitaire des Aliments; Maisons-Alfort, France, 2009.
103. European Food Safety Authority; European Centre for Disease Prevention and Control. The European Union Summary Report on Trends and Sources of Zoonoses, Zoonotic Agents and Food-borne Outbreaks in 2012. EFSA J. 2014, 12, 3547.
104. European Food Safety Authority; European Centre for Disease Prevention and Control. The European Union Summary Report on Trends and Sources of Zoonoses, Zoonotic Agents and Food-borne Outbreaks in 2013. EFSA J. 2015, 13, 3991.
105. European Food Safety Authority; European Centre for Disease Prevention and Control. The European Union Summary Report on Trends and Sources of Zoonoses, Zoonotic Agents and Food-borne Outbreaks in 2014. EFSA J. 2015, 13, 4329.
106. Réveillaud, É.; Desvaux, S.; Boschiroli, M.L.; Hars, J.; Faure, É.; Fediaevsky, A.; Cavalerie, L.; Chevalier, F.; Jabert, P.; Poliak, S.; et al. Infection of wildlife by Mycobacterium bovis in France assessment through a national surveillance system, Sylvatub. Front. Vet. Sci. 2018, 5, 262.

107. Fink, M.; Schleicher, C.; Gonano, M.; Prodinger, W.M.; Pacciarini, M.; Glawischnig, W.; Ryser-Degiorgis, M.P.; Walzer, C.; Stalder, G.L.; Lombardo, D.; et al. Red deer as maintenance host for bovine tuberculosis, Alpine region. Emerg. Infect. Dis. 2015, 21, 464.
108. Moser, I.; Schettler, E.; Hotzel, H.; Herzog, S.; Frölich, K. Mycobacterial infections in free-living cervids in Germany (2002–2006). J. Wildl. Dis. 2011, 47, 999–1004.
109. INSEE. Statistics Agricoles. Available online: https://www.insee.fr/fr/accueil (accessed on 15 December 2017).
110. Griffin, J.F.T.; Mackintosh, C.G. Tuberculosis in deer: Perceptions, problems and progress. Vet. J. 2000, 160, 202–219.
111. ONCFS. Tableaux de Chasse Par Depártment. Réseau "Ongulés Sauvages ONCFS/FNC/FDC". Available online: www.oncfs.gouv.fr/Grands-ongules-Tableaux-de-chasse-ru248/Grands-ongules-Tableaux-de-chasse-departementaux-ar1480 (accessed on 27 November 2018).
112. Statista. Jahresstrecken von Schwarzwild (Wildschweine) in Deutschland von 1997/98 bis 2015/16. Available online: https://de.statista.com/statistik/daten/studie/157728/umfrage/jahresstrecken-von-schwarzwild-in-deutschland-seit-1997-98/ (accessed on 15 December 2017).
113. Maillard, D.; Gaillard, J.M.; Hewison, M.; Ballon, P.; Duncan, P.; Loison, A.; Toïgo, C.; Baubet, E.; Bonenfant, C.; Garel, M.; et al. Ungulates and their management in France. In European Ungulates and Their Management in the 21st Century; Apollonio, M., Andersen, R., Putman, R., Eds.; Cambridge University Press: Cambridge, UK, 2010.
114. Wotschikowski, U. Ungulates and their management in Germany. In European Ungulates and Their Management in the 21st Century; Apollonio, M., Andersen, R., Putman, R., Eds.; Cambridge University Press: Cambridge, UK, 2010.
115. ANSES. Tuberculose Bovine et Faune Sauvage. Rapport Scientifique. Available online: https://www.anses.fr/fr/system/files/SANT2010sa0154Ra.pdf (accessed on 12 December 2018).
116. Rigaux, P.; Chanu, C. Densité du blaireau d'Eurasie (Meles meles) et répartition des terriers dans un paysage rural du Massif Central (Puy-de-Dôme, France). Rev. Ecol. 2012, 67, 339–347.
117. Keuling, O.; Greiser, G.; Grauer, A.; Strauß, E.; Bartel-Steinbach, M.; Klein, R.; Wenzelides, L.; Winter, A. The German wildlife information system (WILD): Population densities and den use of red foxes (Vulpes vulpes) and badgers (Meles meles) during 2003–2007 in Germany. Eur. J. Wildl. Res. 2011, 57, 95–105.
118. C. Sanz. Programa de Erradicación de Tuberculosis Caprina en la Comunidad Autónoma de Extremadura; VIII Foro Nacional del Caprino, Servicio de Sanidad Animal, Junta de Extremadura; 2017. Available online: https://www.cabrandalucia.com/inicio/foro-nacional-caprino/viii-foro-nacional-del-caprino (accessed on 12 December 2018).
119. Gortázar, C.; Fernández-Calle, L.M.; Collazos-Martínez, J.A.; Mínguez-González, O.; Acevedo, P. Animal tuberculosis maintenance at low abundance of suitable wildlife reservoir hosts: A case study in northern Spain. Prev. Vet. Med. 2017, 146, 150–157.
120. Fernández-de-Mera, I.G.; Vicente, J.; Höfle, U.; Ruiz-Fons, F.; Ortiz, J.A.; Gortázar, C. Factors affecting red deer skin test responsiveness to bovine and avian tuberculin and to phytohaemagglutinin. Prev. Vet. Med. 2009, 90, 119–126.
121. Vicente, J.; Barasona, J.A.; Acevedo, P.; Ruiz-Fons, J.F.; Boadella, M.; Diez-Delgado, I.; Beltran-Beck, B.; González-Barrio, D.; Queirós, J.; Montoro, V.; et al. Temporal trend of tuberculosis in wild ungulates from Mediterranean Spain. Transbound. Emerg. Dis. 2013, 60, 92–103.
122. García-Jiménez, W.L.; Fernández-Llario, P.; Benítez-Medina, J.M.; Cerrato, R.; Cuesta, J.; García-Sánchez, A.; Gonçalves, P.; Martínez, R.; Risco, D.; Salguero, F.J.; et al. Reducing Eurasian wild boar (Sus scrofa) population density as a measure for bovine tuberculosis control: Effects in wild boar and a sympatric fallow deer (Dama dama) population in Central Spain. Prev. Vet. Med. 2013, 110, 435–446.
123. García-Bocanegra, I.; de Val, B.P.; Arenas-Montes, A.; Paniagua, J.; Boadella, M.; Gortázar, C.; Arenas, A. Seroprevalence and risk factors associated to Mycobacterium bovis in wild artiodactyl species from southern Spain, 2006–2010. PLoS One 2012, 7, e34908.
124. De Val, B.P.; Napp, S.; Velarde, R.; Lavín, S.; Cervera, Z.; Singh, M.; Allepuz, A.; Mentaberre, G. Serological follow-up of tuberculosis in a wild boar population in contact with infected cattle. Transbound. Emerg. Dis. 2017, 64, 275–283.

125. Santos, N.; Nunes, T.; Fonseca, C.; Vieira-Pinto, M.; Almeida, V.; Gortázar, C.; Correia-Neves, M. Spatial analysis of wildlife tuberculosis based on a serologic survey using dried blood spots, Portugal. Emerg. Infect. Dis. 2018, 24, 2169–2175.
126. Madeira, S.; Manteigas, A.; Ribeiro, R.; Otte, J.; Fonseca, A.P.; Caetano, P.; Abernethy, D.; Boinas, F. Factors that influence Mycobacterium bovis infection in red deer and wild boar in an epidemiological risk area for tuberculosis of game species in Portugal. Transbound. Emerg. Dis. 2015, 64, 793–804.
127. Muñoz-Mendoza, M.; Marreros, N.; Boadella, M.; Gortázar, C.; Menéndez, S.; de Juan, L.; Bezos, J.; Romero, B.; Copano, M.F.; Amado, J.; et al. Wild boar tuberculosis in Iberian Atlantic Spain: A different picture from Mediterranean habitats. BMC Vet. Res. 2013, 9, 176.
128. Barasona, J.A.; Mulero-Pázmány, M.; Acevedo, P.; Negro, J.J.; Torres, M.J.; Gortázar, C.; Vicente, J. Unmanned aircraft systems for studying spatial abundance of ungulates: Relevance to spatial epidemiology. PLoS One 2014, 9, e115608.
129. Balseiro, A.; Rodríguez, O.; González-Quirós, P.; Merediz, I.; Sevilla, I.A.; Davé, D.; Dalley, D.J.; Lesellier, S.; Chambers, M.A.; Bezos, J.; et al. Infection of Eurasian badgers (Meles meles) with Mycobacterium bovis and Mycobacterium avium complex in Spain. Vet. J. 2011, 190, e21–e25.
130. MAGRAMA. Caracterización del Sector Ovino y Caprino en España, año 2016. In Ministerio de Agricultura, Alimentación y Medio Ambiente, Dirección General de Producciones y Mercados Agrarios, Subdirección General de Productos Ganaderos; Technical Report; 2015. Available online: https://www.mapa.gob.es/es/ganaderia/temas/produccion-y-mercados-ganaderos/caracterizaciondelsectorovino2016_def_tcm30-380879.pdf (accessed on 12 December 2018).
131. Armenteros, J.A.; Barasona, J.Á.; Boadella, M.; Acevedo, P.; Gortázar, C.; Vicente, J. Una propuesta para considerar aspectos sanitarios en la regulación cinegética. Rev. Ecosist. 2013, 22, 54–60.
132. MAGRAMA. Anuarios de Estadística Forestal. Available online: https://www.miteco.gob.es/es/biodiversidad/estadisticas/5016%20Estad%C3%ADstica%20Anual%20de%20Caza_METODOLOG%C3%8DA_tcm30-196241.pdf (accessed on 14 November 2018).
133. ICNF. Hunting Statistics Database Portugal; Instituto de Conservação da Natureza e Biodiversidade: 2016 (accessed on 29 March 2019).
134. Vingada, J.; Fonseca, C.; Cancela, J.; Ferreira, J.; Eira, C. Ungulates and their management in Portugal. In European Ungulates and Their Management in the 21st Century; Apollonio, M., Andersen, R., Putman, R., Eds.; Cambridge University Press: Cambridge, UK, 2010.
135. Carranza, J. Ungulates and their management in Spain. In European Ungulates and Their Management in the 21st Century; Apollonio, M., Andersen, R., Putman, R., Eds.; Cambridge University Press: Cambridge, UK, 2010.
136. Rosalino, L.M.; Macdonald, D.W.; Santos-Reis, M. Spatial structure and land-cover use in a low-density Mediterranean population of Eurasian badgers. Canad. J. Zool. 2004, 82, 1493–1502.
137. Revilla, E.; Delibes, M.; Travaini, A.; Palomares, F. Physical and population parameters of Eurasian badgers (Meles meles L.) from Mediterranean Spain. Z. Säugetierkd. 1999, 64, 269–276.
138. Acevedo, P.; González-Quirós, P.; Prieto, J.M.; Etherington, T.R.; Gortázar, C.; Balseiro, A. Generalizing and transferring spatial models: A case study to predict Eurasian badger abundance in Atlantic Spain. Ecol. Model. 2014, 275, 1–8.
139. EEA. Biogeographical Regions of Europe. Council of Europe (CoE), Directorate-General for Environment (DG ENV), European Environmental Agency. Available online: www.eea.europa.eu/legal/copyright (accessed on 15 December 2017).
